# The Relationship of Dry Eye Disease With Depression in Saudi Arabia: A Cross-Sectional Study

**DOI:** 10.7759/cureus.12160

**Published:** 2020-12-18

**Authors:** Walaa Al-Dairi, Omar M AL Sowayigh, Noura S Alkulaib, Ali Alsaad

**Affiliations:** 1 Surgery, College of Medicine, King Faisal University, AlAhsa, SAU; 2 Ophthalmology, College of Medicine, King Faisal University, AlAhsa, SAU; 3 Psychiatry, College of Medicine, King Faisal University, AlAhsa, SAU

**Keywords:** dry eye disease, dry eye disorder, depression, patient health questionnaire (phq-9), dry eye questionnaire (deq-5), contact lens

## Abstract

Objective

Estimate the prevalence of depression among individuals with a dry eye disease (DED) in Saudi Arabia using two questionnaires: Patient Health Questionnaire (PHQ-9) and Dry Eye Questionnaire (DEQ-5), and explore potential factors implicated in the development of depression among the DED population.

Methods

This is a descriptive cross-sectional study of 476 patients with DED which was conducted using a PHQ-9 questionnaire to screen for depression and a DEQ-5 questionnaire to diagnose DED. The questionnaires were merged and distributed using Google Forms through various social media platforms targeting the Saudi population. After data collection, it was revised, coded and fed to statistical software IBM SPSS version 22 (SPSS, Inc. Chicago, IL).

Results

Depression was diagnosed among 200 participants (42%) of the cases with dry eyes. From which 5.7% had mild depression, 13.9% had moderate depression, 12.6% had moderately severe depression, and 9.9% had severe depression. A female predominance was noticed; 44.7% of the females with dry eyes had depression compared to 32.4% of males with recorded statistical significance (P=0.023). Depression was detected among 55% of those who are less than 20 years old in comparison to 27% of those who are 30 years or older (P=0.001). Laser-assisted in-situ keratomileusis (LASIK), prolonged electronic device usage and contact lens wear are reported as risk factors associated with an increase in dry eye symptoms. However, there is no statistically significant relationship between contact lens wear and depression among dry eye disease patients.

Conclusion

Suffering from DED is a possible risk factor for developing depression as DED is associated with depression of higher degrees of severity. Depression among DED patients is found to be significantly more prevalent among females and the young adult population rather than older adults.

## Introduction

Dry eye disease (DED) is a multifactorial disease that affects the tear film and ocular surface of the eye. DED leads to abnormal alterations of the normal ocular surface physiology including an increase in the osmolarity of the tear film and inflammation. As a result of these changes, patients with dry eyes complain of eye discomfort, irritation or burning sensation in addition to visual disturbances. Furthermore, tear film instability is noted on tear breakup time with potential damage to the ocular surface [1].

DED has a worldwide prevalence of 5-50% [2]. In Saudi Arabia, the prevalence is high yet highly variable, which is owed to the different studied provinces and cities ranging from 32.1% to 93.2% [3-5]. Globally, DED is more prevalent among females [2,6], with a similar finding in Saudi Arabia [3]. Other than female gender predominance, risk factors include old age [7], blepharitis [5], smoking [5] and having a chronic disease such as diabetes mellitus, arthritis and hypercholesterolemia [7,8].

DED has a significant impact on both the patient's quality of life, noted by Short Form 36 Health Survey Questionnaire (SF-36) scores [9], and vision-related quality of life, noted by difficulties in reading, watching television, conducting professional work, computer use and driving [10]. Furthermore, DED negatively influences psychological health, and in effect, is associated with developing depression [11,12]. Depression is a serious, however, overlooked mental health illness that negatively affects the mood and is potentially life-threatening if neglected. Although the etiology is still poorly understood, numerous factors and diseases have been linked to or resulted in depression.

Among other eye diseases, DED was found to be the only disease to have an increased depression score using Zung’s self-rated depression scale [12]. Many studies in the literature have found a higher prevalence of depression among DED patients compared to controls [13] and a linear association between DED and depressive symptoms [14] which suggests a higher risk among chronic severe DED patients [11,14]. The visual impairment caused by dry eye symptoms is directly linked to depressive symptoms [15]. Furthermore, a reverse relationship was reported as well, in which depression was associated with DED [16] and worsening of DED symptoms [14]. In this respect, DED and depression have a manifest interrelation in ways that have a negative impact on the patients [17].

Depression screening questionnaires and self-rating scales such as the nine-item Patient Health Questionnaire (PHQ-9) and Zung’s self-rated depression scale have been useful in primary care settings and research purposes [18,19]. These scales have also been used in studies evaluating the relationship between certain eye diseases and depression [12,20]. Applying the same concept, the Dry Eye Questionnaire (DEQ-5) has been used in the diagnosis of DED with quantification of its severity level [21,22].

Despite the high prevalence of both DED and depression in Saudi Arabia [3-5,23], the psychological impact of DED has not been studied adequately. Therefore, this study aims to evaluate the relationship between DED and depression in Saudi Arabia using two questionnaires: PHQ-9 and DEQ-5.

## Materials and methods

Study design and participants

This descriptive cross-sectional study was conducted using a PHQ-9 questionnaire to screen for depression and a DEQ-5 questionnaire to diagnose DED. DEQ-5 was translated into Arabic for easy accessibility to the targeted population (Appendices). A unified single questionnaire was distributed using Google Forms through various social media platforms targeting Saudi individuals.

This study included participants who are aged 15 and older, who are either previously diagnosed with dry eyes or had DED based on DEQ-5. For exclusion criteria, all individuals who were previously diagnosed with depression or suffering from any other psychiatric illness, have more than one comorbid condition (e.g., heart disease, hypertension, diabetes mellitus, asthma, etc.), were grieving the death of a close person, or had a recent ocular procedure done in less than a year including laser-assisted in-situ keratomileusis (LASIK), crosslinking or corneal transplantation were excluded. Among 1568 participants, only 476 (30.35%) met the criteria and were included for analysis.

Other data include biographical information such as age, gender, province/city, eye conditions, eye procedures, daily screen time, and contact lens use.

Statistical analysis

After data were extracted, it was revised, coded, and fed to statistical software IBM SPSS version 22 (SPSS, Inc. Chicago, IL). All statistical analysis was done using two-tailed tests. A P-value less than 0.05 was statistically significant. Descriptive analysis based on frequency and percent distribution was done for patient’s characteristics, eye care, and depression severity. Regarding PHQ-9, the total score for the different items was summed and categorized into no depression (zero score), minimal depression (1-4), mild depression (5-9), moderate depression (10-14), moderately severe depression (15-19), and severe depression (20-27). Cross-tabulation was used to assess the relationship between personal data and depression and between eye care and dry eye data with different items including contact lens use, daily screen time. Pearson chi-square and exact probability tests were used to test for distribution difference significance.

Ethical consideration

This study was conducted upon the approval of the research ethics committee of the College of Medicine, King Faisal University. The DEQ-5 questionnaire was used with direct permission from Robin L Chalmers. All data were confidentially and anonymously collected with consent taken from the participants.

## Results

This study included 476 cases with dry eyes whose ages ranged from 15 to 54 years old with a mean age of 22.7 ± 6.8 years old. The majority of the participants were females (77.9%; 371) and 212 (44.5%) work indoors with prolonged electronic screen using. Regarding regions, 155 cases (32.6%) were from Riyadh and 70 (14.7%) were from Al-Ahsa. Over 235 cases (49.4%) were previously diagnosed with dry eyes and 50.6% were diagnosed using the DEQ-5 scale. Also, 63 (13.2%) had a chronic health problem (e.g., heart disease, hypertension, diabetes mellitus, asthma, etc.). As for other eye diseases, astigmatism was the most reported by the cases (81.4%; 79), followed by myopia (76.3%; 74), hyperopia (29.9%; 29), and amblyopia (12.4%; 12) while only three cases had glaucoma (3.1%; Table 1).

**Table 1:** Personal data of patients with DED, Saudi Arabia, 2020. DED: dry eye disease, DEQ: Dry Eye Questionnaire.

Personal data		No	%
Gender	Male	105	22.1
	Female	371	77.9
Age	<20 years	180	37.8
	20-29 years	233	48.9
	30+ years	63	13.2
Job/study type	Indoor+ does not use electronic screens	143	30.0
	Indoor + high electronic screens usage	212	44.5
	Jobless - retired	86	18.1
	Outdoor + does not use electronic screens	14	2.9
	Outdoor + high electronic screen using	21	4.4
Region	Al Bahah region	6	1.3
	Dhahran	8	1.7
	Jouf region	3	0.6
	Al-Ahsa	70	14.7
	Jubail	7	1.5
	Khobar	4	0.8
	Riyadh	155	32.6
	Taif	16	3.4
	Almadinah almunawarah	26	5.5
	Aseer region	14	2.9
	Dammam	23	4.8
	Hafar Albatin	9	1.9
	Hail region	12	2.5
	Jazan region	7	1.5
	Jeddah	38	8.0
	Makkah	21	4.4
	Najran region	3	0.6
	Northern borders region	3	0.6
	Qassim region	37	7.8
	Qatif	6	1.3
	Tabuk region	8	1.7
Dry eye	Diagnosed on DEQ-5	241	50.6
	Previously diagnosed	235	49.4
Comorbid diseases	No	413	86.8
	Yes	63	13.2
Other eye diseases	Astigmatism	79	81.4
	Myopia	74	76.3
	Hyperopia	29	29.9
	Amblyopia	12	12.4
	Keratoconus	1	1.0
	Conjunctivitis	8	8.2
	Cataract	1	1.0
	Glaucoma	3	3.1
	Others	3	3.1

Depression was diagnosed among 200 (42%) of the cases with dry eyes, of which 5.7% had mild depression, 13.9% had moderate depression, 12.6% had moderately severe depression, and 9.9% had severe depression (Table 2).

**Table 2:** Prevalence of depression and its severity among patients with dry eye disease.

Depression severity	No	%
No depression	276	58.0
Depression	200	42.0
Mild depression	27	5.7
Moderate depression	66	13.9
Moderately severe depression	60	12.6
Severe depression	47	9.9

Regarding gender predilection, 44.7% of females with DED had depression compared to 32.4% of males with recorded statistical significance (P=0.023). As for age, depression was detected among 55% of those who were less than 20 years of age in comparison to 27% of those who were 30 years or older (P=0.001). Considering the geographical distribution of depression in our sample, there was percentage variability among the regions, however, a larger sample size would help to eliminate any statistical bias.

Considering other concurrent eye diseases, 66.7% of those with amblyopia had depression compared to 62.5% of those with conjunctivitis and 51.9% of those with astigmatism (P=0.379). An exact 42.6% of those with no health problem were depressed compared to 38.1% of those with chronic health problem with no statistical significance (Table 3).

**Table 3:** Effect of different patients with dry eye disease characteristics and comorbidities on depression status. P: Pearson X2 test, N/A: not applicable. *P<0.05 (significant). ^$^Exact probability test.

Bio-medical data		Final diagnosis				P-value
		Depressed		Not depressed		
		No	%	No	%	
Gender	Male	34	32.4	71	67.6	0.023*
	Female	166	44.7	205	55.3	
Age	< 20 years	99	55.0	81	45.0	0.001*
	20-29 years	84	36.1	149	63.9	
	30+ years	17	27.0	46	73.0	
Region	Al Bahah region	3	50.0	3	50.0	0.044*^$^
	Dhahran	8	100.0	0	0.0	
	Jouf region	0	0.0	3	100.0	
	Al-Ahsa	20	28.6	50	71.4	
	Jubail	2	28.6	5	71.4	
	Khobar	1	25.0	3	75.0	
	Riyadh	62	40.0	93	60.0	
	Taif	5	31.3	11	68.8	
	Almadinah almunawarah	15	57.7	11	42.3	
	Aseer region	6	42.9	8	57.1	
	Dammam	11	47.8	12	52.2	
	Hafar albatin	3	33.3	6	66.7	
	Hail region	6	50.0	6	50.0	
	Jazan region	4	57.1	3	42.9	
	Jeddah	17	44.7	21	55.3	
	Makkah	12	57.1	9	42.9	
	Najran region	0	0.0	3	100.0	
	Northern borders region	1	33.3	2	66.7	
	Qassim region	15	40.5	22	59.5	
	Qatif	4	66.7	2	33.3	
	Tabuk region	5	62.5	3	37.5	
Dry eye	Diagnosed on DEQ-5	120	49.8	121	50.2	N/A
	Previously diagnosed	80	34.0	155	66.0	
Other eye diseases	Astigmatism	41	51.9	38	48.1	0.379^$^
	Myopia	37	50.0	37	50.0	
	Hyperopia	12	41.4	17	58.6	
	Amblyopia	8	66.7	4	33.3	
	Keratoconus	0	0.0	1	100.0	
	Conjunctivitis	5	62.5	3	37.5	
	Cataract	0	0.0	1	100.0	
	Glaucoma	1	33.3	2	66.7	
	Others	0	0.0	3	100.0	
Have chronic diseases	No	176	42.6	237	57.4	0.498
	Yes	24	38.1	39	61.9	

Table 4 illustrates the daily screen time and history of eye procedures among patients with dry eye. A small percentage (12.6%) recorded daily screen time of less than five hours while 33.2% recorded 10 hours or more. An increase of dry eye symptoms associated with electronic device usage was reported by 413 (86.8%) of the cases. With respect to ocular procedures, 7.8% had a procedure more than a year. LASIK surgery was the most reported procedure (5.9%; 28) followed by the chalazion procedure (1.7%; 8). Daily contact lens (CL) use was reported by 17 cases (3.6%). An increase in dryness symptoms with CL use was reported by 42% of the cases while 44.6% reported that it may occur sometimes. The need of using artificial eye drops during CL wear was reported by 86 (76.8%) of the cases, and 64 (58%) became intolerant of CL use which pushed 53 (82.8%) to use eyeglasses.

**Table 4:** Daily screen time and history of eye procedures among patients with dry eye disease. CL: contact lens, LASIK: laser-assisted in-situ keratomileusis.

Eye procedures and screen time data		No	%
Daily screen time	<5 hours	60	12.6
	5-9 hours	258	54.2
	10+ hours	158	33.2
Dry eye symptoms increase with electronic devices usage	Yes	413	86.8
	No	63	13.2
Had an eye procedure	No	434	91.2
	Yes, less than a year	5	1.1
	Yes, more than a year	37	7.8
Type of procedure	None	434	91.2
	Chalazion procedure	8	1.7
	LASIK	28	5.9
	Punctual occlusion	1	0.2
	Removal of foreign object procedure	1	0.2
	Retinal detachment procedure	1	0.2
	Retinal procedure	1	0.2
	Strabismus procedure	2	0.4
CL use	Daily	17	3.6
	1-2 days/ week	14	2.9
	Sometimes	81	17.0
	No	364	76.5
Dryness increases with CL use	Yes	47	42.0
	Sometimes	50	44.6
	No	15	13.4
Use of eye drops during CL wear	Yes	86	76.8
	No	26	23.2
Became intolerant to CL use	Yes	64	58.0
	No	47	42.0
If yes, does it push you to use eyeglasses?	Yes	53	82.8
	No	11	17.2

Depression was detected among 47.1% of those who use CL daily in comparison to 59.3% of those who did not use it (P=0.481; Table 5). Intolerance to CL developed in 52.3% of the wearers compared to 55.3% of those who were able to continue wearing them (P=0.752). Also, 52.8% of those who have dry eyes and used eyeglasses for refractive errors had depression compared to 45.5% of those who did not need to wear glasses (P=0.656).

**Table 5:** Distribution of depression among patients with dry eyes according to CL use. CL, contact lens, P: Pearson X^2^ test. ^$^Exact probability test.

		Final diagnosis				P-value
		Depressed		Not depressed		
		No	%	No	%	
CL use	Daily	9	52.9	8	47.1	0.481
	1-2 days/week	8	57.1	6	42.9	
	Sometimes	35	43.2	46	56.8	
	No	148	40.7	216	59.3	
	No	15	57.7	11	42.3	
Became intolerant of CL use	Yes	31	47.7	34	52.3	0.752
	No	21	44.7	26	55.3	
If yes, does it push you to use eyeglasses?	Yes	25	47.2	28	52.8	0.656^$^
	No	6	54.5	5	45.5	

## Discussion

To the best of our knowledge, this is the first study to explore the relationship between DED and depression in Saudi Arabia. Using PHQ-9 and DEQ-5 questionnaires, we were able to assess depression and DED among Saudi adults living in Saudi Arabia. We found that 42% of individuals suffering from dry eyes had depression with various degrees of severity. Those individuals were not previously diagnosed with depression or any psychiatric illness nor were in a grief state. By eliminating such conditions, we emphasized the impact of DED on the quality of life and mood.

Similar studies have explored the associations of DED, depression and anxiety. Most of these studies have proved a significant relationship [13,17,24]. According to the literature, the relationship seems to start either way, where DED plays a role in experiencing depression and vice versa [17]. This signifies the importance of detecting depression among individuals with DED as well as identifying the role of chronic conditions and their medications which could be the underlying cause for dry eyes among many individuals.

It has been previously established that depression is more prevalent among females [2,6], which is a similar finding in this study where females with DED were significantly more likely to suffer from depression than males. We also found that young adults with DED tend to suffer from depression more than older adults. This is in accordance with other studies that found depression to be less common among older adults [25]. Although the depression rate among DED patients (42%) is not higher than the rate reported in 2014 among the general Saudi adult population (49.9%) [23], depression's severity is higher among DED patients.

Having a concurrent eye disease did not seem to have a significant relationship with developing depression. This is clearly evident in the investigated common ocular conditions like astigmatism, myopia, and hyperopia. However, a wider study scope with a larger inclusive sample will be needed to investigate the other ocular pathologies mentioned in our study which are: amblyopia, keratoconus, conjunctivitis, cataract, and glaucoma. Having a concomitant chronic disease seemed to has no effect on developing depression among individuals with DED. We assume that the direct impact of DED on the person’s daily activity is the reason behind the tendency of developing depression by the affected individuals more than other chronic health conditions with subtle psychological effects.

Furthermore, Akkaya et al. [26] described a comparable finding to this study where DED symptoms increase with prolonged daily electronic device usage. Among the different eye procedures, LASIK and cataract surgeries were the most procedures known to result in transient DED as reported in the literature [27,28]. These findings point out the role of ocular procedures and individual’s screen time in developing DED. LASIK surgery is complicated by DED mainly because of iatrogenic corneal nerve damage [29]. On the other hand, the use of electronic devices decreases the number of eye blinks and subsequently leads to dry eye symptoms [26]. Hence, recognizing this fact should allow individuals to exercise a protective attitude against DED and its consequences by improving lifestyle, such as limiting screen time as much as possible, use of 20-20-20 rule to reduce the symptoms of eye strain and dryness associated with prolonged electronic device usage, enhancing the indoor humidity by using a humidifier and reducing or stopping CL wear if DED is active.

Although we report an increase in DED symptoms among CL wearers, as reported in the literature [30], there was not a significant relationship between CL use and depression among DED patients. This may be attributed to the fact that there are some individual’s behaviors such as CL could increase the DED symptoms in DED patients. However, these behaviors have not increased the risk of developing depression.

A limitation of the study was the use of social media to collect responses, and as a result, most of the participants were mainly of the young age group. Although the scales used in our study have been validated [18,22], they are more aimed at screening rather than diagnosis, and therefore, this may overestimate the true prevalence. Participants may have been untruthful and biases cannot be excluded. A larger sample size with the inclusion of other ocular comorbidities can shed light on the psychological impact of ocular pathology on individuals’ mental wellbeing.

## Conclusions

In conclusion, suffering from DED is a possible risk factor for developing depression as DED is associated with depression of higher degrees of severity. Depression among DED patients is found to be significantly more prevalent among females and young adult population rather than older adults.

We recommend screening for depression among patients diagnosed with DED in ophthalmology clinics as well as primary care clinics as a proactive measure in improving life quality and to preserve their psychological well-being.

## Figures and Tables

**Figure 1:**
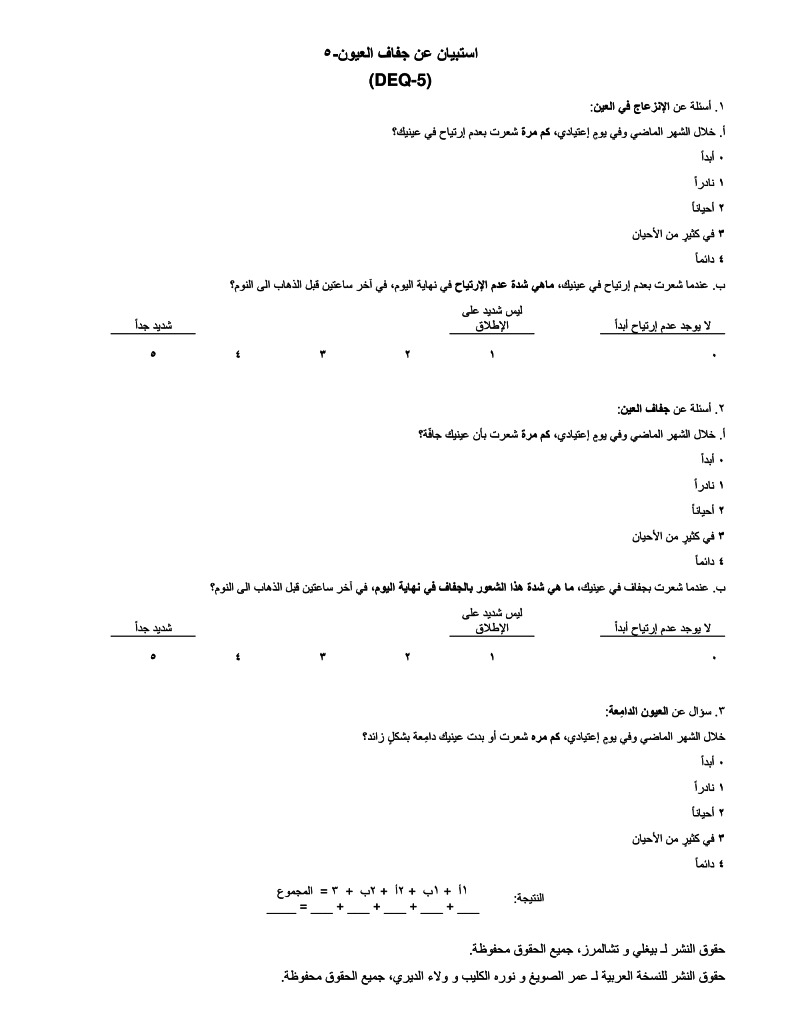
Arabic version of DEQ-5
